# Geniposide suppresses NLRP3 inflammasome-mediated pyroptosis via the AMPK signaling pathway to mitigate myocardial ischemia/reperfusion injury

**DOI:** 10.1186/s13020-022-00616-5

**Published:** 2022-06-17

**Authors:** Haiyan Li, Dong-Hua Yang, Yanmei Zhang, Fuchun Zheng, Fenfei Gao, Jiajia Sun, Ganggang Shi

**Affiliations:** 1 Department of Pharmacology, Shantou University Medical College, Shantou, 515041 China; 2 Department of Pharmaceutical Sciences, College of Pharmacy and Health Sciences, St. John’s University, Queens, NY 11439 USA; 3 Reproductive Center of the First Affiliated Hospital of Shantou University Medical College, Shantou, 515000 Guangdong China

**Keywords:** Myocardial ischemia reperfusion, Geniposide, AMPK, NLRP3 inflammasome, Pyroptosis

## Abstract

**Background:**

NLRP3 inflammasome activation and pyroptosis play a significant role in myocardial ischemia reperfusion injury (MI/RI). Geniposide was reported to show potential therapeutic use for MI/RI with its anti-inflammatory and anti-oxidative properties. However, research on the specific mechanism of geniposide has not been reported.

**Methods:**

The MIRI model of animal was created in male C57BL/6J mice and the hypoxia reoxygenation (H/R) model was established for the in vitro experiments. Neonatal rat ventricular myocytes (NRVMs) and H9c2 cells with knockdown of TXNIP or NLRP3 were used. Geniposide was administered to mice before vascular ligation. HE staining, 2,3,5-triphenyltetrazolium chloride (TTC) staining, echocardiography, oxidative stress and myocardial enzyme detection were used to evaluate the cardioprotective effect of geniposide. Meanwhile, pharmacological approaches of agonist and inhibitor were used to observe potential pathway for geniposide cardioprotective in vitro and in vivo. Moreover, ELISA kits were adopted to detect the levels of inflammatory factors, such as IL-1β and IL-18. The gene and protein expression of NLRP3 and pyroptosis-related factors in heart tissue were performed by RT-PCR, western blotting and immunofluorescence in vivo and in vitro, respectively.

**Results:**

Our results indicate that geniposide can reduce the area of myocardial infarction, improve heart function, and inhibit the inflammatory response in mice after MI/RI. In addition, RT-PCR and western blotting shown geniposide promoting AMPK phosphorylation to activate myocardium energy metabolism and reducing the levels of genes and proteins expression of NLRP3, ASC, N-GSDMD and cleaved caspase-1, IL-1β, IL-18. Meanwhile, geniposide improved NRVMs energy metabolism, which decreased ROS levels and the protein expression of TXNIP and thus suppressed the expression of NLRP3. AMPK antagonist or agonist and siRNA downregulation of TXNIP or NLRP3 were also verify the effect of geniposide against H/R injury. Further research found that geniposide promoted the translocation of TXNIP and reduce the binding of TXNIP and NLRP3.

**Conclusions:**

In our study, geniposide can significantly inhibit NLRP3 inflammasome activation via the AMPK signaling pathway and inhibit pyroptosis of cardiomyocytes in myocardial tissues.

**Supplementary Information:**

The online version contains supplementary material available at 10.1186/s13020-022-00616-5.

## Background

Myocardial ischemia/reperfusion injury (MI/RI) is involved in various pathological states. Clinically, some patients have blood pressure instability, severe arrhythmia, deterioration of ventricular systolic function and even sudden death, which obviously affect the life of patients [1]. Increasing myocardial cell death and aggravating myocardial infarction are the most serious consequences of ischemia–reperfusion injury. Despite of many years of efforts to reduce ischemia–reperfusion injury, MI/RI-associated death rate remains persistently high. Therefore, it is important to determine the mechanism of coronary artery disease progression and establish methods to protect the myocardium from MI/RI damage.

Adenosine monophosphate-activated protein kinase (AMPK) regulates the balance of cell energy metabolism and intracellular stress in eukaryotic cells, due to its crucial role in controlling energy homeostasis. Previous studies have shown that AMPK is a potential target of various human diseases [2, 3]. In the cardiovascular system, the relationship between AMPK and ischemic diseases has been studied for decades. Various of studies have found that AMPK activation confer a protective benefit in cardiomyocyte under MI/RI by lessening cell apoptosis, improving post-ischemic recovery, and reducing myocardial infarction [4, 5].

Inflammatory bodies play a crucial role in immunity and Inflammation. The nucleotide-binding domain, leucine-rich repeat containing receptor (NLR) proteins, which consist of pyrin domain-containing (NLRP) proteins NLRP1 and NLRP3 and CARD-containing (NLRC) receptor NLRC4, belong to the intracellular pathogen family and danger signal sensors and are part of the innate immunity effector molecules [6]. They can recruit the effector procaspase-1 and CARD, the apoptosis-associated speck-like protein, to form a series of multimeric protein complexes [7]. The activation of inflammasome can further induce pyroptosis. Pyroptosis is a type of inflammatory programmed cell death closely correlated with NLRP3 inflammasome including NLRP3, ASC, caspase-1, and N-GSDMD proteins [8]. The activation of NLRP3 inflammasome and gasdermin D (GSDMD), instead of apoptotic caspase, is essential for inducing cell pyroptosis which is manifested as DNA damage, cell swelling, formation of cell membrane pores and release of cellular contents. Its occurrence depends on the activation of inflammatory factors. Caspase-1 is an NLRP3 inflammatory effector [9]. In the cell membrane, caspase-1-activated GSDMD can produce N-terminal fragment (N-GSDMD) oligomers, leading to rupture of the cell membrane, causing cellular pyroptosis and forming larger membrane pores. Meanwhile, caspase-1 activation can process the IL-1β and IL-18 precursors to mature IL-1β and IL-18, thus inducing inflammation [10, 11].

At present, substantial evidence indicates that NLRP3 inflammasome-mediated pyroptosis originated from NLRP3 inflammasome and activated caspase-1, as well as the maturation and secretion of IL-1β and IL-18 after MI/RI insult, which is greatly participated in the pathological process of MI/RI [12]. Furthermore, activated NLRP3 inflammasome aggravates myocardial injury by directly initiating caspase-1-mediated pyroptosis and indirectly inducing the release of pro-inflammatory mediators. However, inhibiting the pyroptosis mediated by NLRP3 inflammasome can decreased myocardial infarcted size and therefore restore the cardiac function of mice [13]. Therefore, suppressing the activation of NLRP3 inflammasome and pyroptosis exert the benefit for alleviating myocardial injury. Recently, it was reported that the NLRP3 inflammasome is induced by thioredoxin-interacting protein (TXNIP) under oxidative stress [14, 15]. In physiological circumstances, as a protein complex related to redox, TXNIP binds to thioredoxin (Trx). Trx is antioxidant defense protein, play an essential role in cell function by participating in redox‐dependent processes by regulating apoptosis and interacting with transcription factors to maintain protein folding [16–18]. as the result, the NLRP3 inflammasome remains inactive due to the abnormal interaction with TXNIP. Oxidation activators induce TXNIP to cleave from thioredoxin and bind to NLRP3. In addition, several studies have reported that TXNIP transfers from the nucleus to the cytoplasm in order to response to oxidative stress [19, 20].

**Fig. 1:**
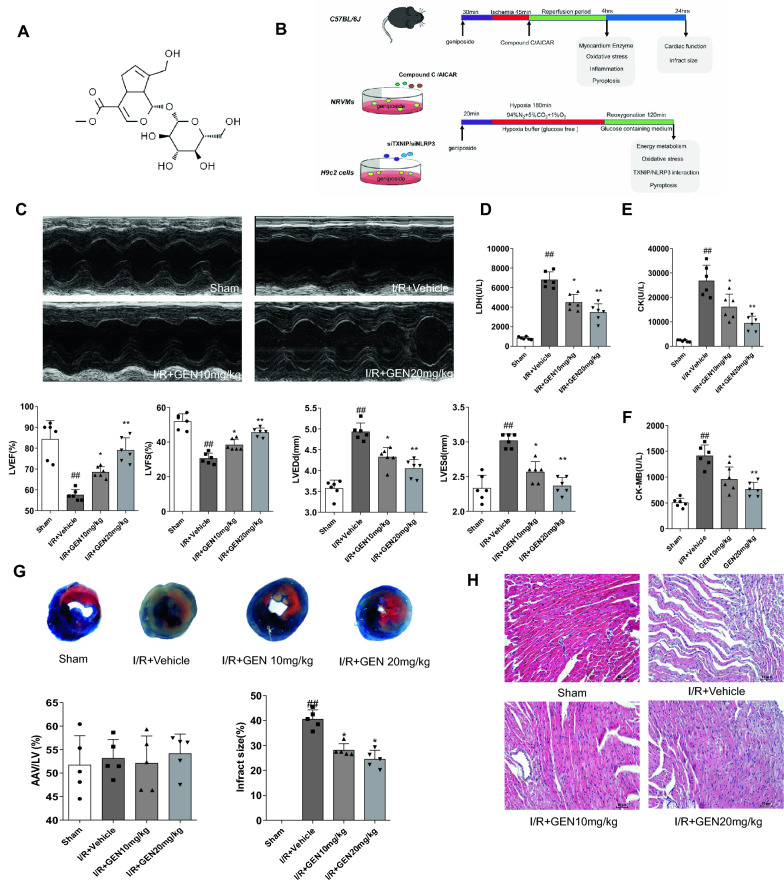
Geniposide improves cardiac function of mice suffering from MI/RI. **A** The chemical structure of geniposide. **B** The experimental design of our work. To examinate the effect of geniposide on MI/RI, the animals were divided into GEN treatment, I/R + Vehicle, and sham groups. To determine the mechanism of action of geniposide, another set of animals was administered with either Compound C or AICAR immediately at the beginning of reperfusion. The H/R model was launched in NRVMs and H9c2 cells. Compound C and AICAR, si-TXNIP, or si-NLRP3 were adopted to reveal the roles of pyroptosis and the AMPK/TXNIP/NLRP3 signaling on the cardioprotective effect of geniposide. **C** Representative echocardiograms in M-mode records of left ventricular (LV) and analysis of LV ejection fraction (LVEF), fractional shortening (LVFS), LV end-diastole diameter (LVEDd), and LV end-systolic diameter (LVESd) (n = 6). **D**–**F** The activity of myocardial enzymes LDH, CK-MB, and CK levels in serum of mice (n = 6). **G** Representative TTC–Evans Blue stained sections of hearts and quantitative data of the LV infarct size. Infarct size (%) was expressed as the percentage of infarct area relative to the total left ventricular area. the nonischemic section shown in blue area, red represent risk area, and the infarct region is stained white (n = 6). **H** Representative H&E staining of the left ventricular area. Scale bar = 50 μm (n = 5). The data for each group were shown as the mean ± SD; ^#^p < 0.05, ^##^p < 0.01 vs. Sham group; *p < 0.05, **p < 0.01 vs. I/R + Vehicle group

Although many descriptions of the use of geniposide in the treatment of cardiovascular diseases have shown decent curative effects [21–23], few studies reported cardioprotective effects against MI/RI. Therefore, we explored the potential protective effects of geniposide on the NLRP3 inflammasome and subsequent cardiomyocyte pyroptosis and the underlying mechanisms in a mouse model of MI/RI. *Gardenia jasminoides* Ellis (Rubiaceae) is a source of a traditional Chinese medicine widely used in practice to treat brain diseases [24], liver disorders [25], inflammation, and contusions [25–27]. Geniposide (Fig. 1A), an iridoid isolated from gardenia fruit, has been used as an antioxidant and antineoplastic drug, and has other pharmacological properties such as anti-asthma and anti-diabetic effects. In addition, research in recent years revealed that geniposide helped in MI/RI, mainly related to inhibition of autophagy [28]. However, the underlying mechanism of geniposide to mitigate MI/RI is not clear, and whether geniposide can improve MI/RI by suppressing the NLRP3 inflammasome and pyroptosis in mice remains unknown.

In our study, we demonstrated that geniposide suppresses the NLRP3 inflammasome mediated pyroptosis in vivo. Moreover, we found that geniposide improved myocardic energy metabolism, alleviated oxidative stress, decreased the NLRP3 inflammasome activity and subsequent pyroptosis level, thereby alleviated MI/RI. Based on these results, we verified the anti-hypoxia reoxygenation(H/R) effect of geniposide in the H/R model of neonatal rat ventricular myocytes (NRVMs). The underlying mechanisms was also investigated, and we discovered that geniposide improved NRVMs energy metabolism, which decreased ROS levels and the protein expression of TXNIP and thus suppressed the expression of NLRP3. Further research found that geniposide promoted the translocation of TXNIP and decreased the binding of TXNIP and NLRP3. Compound C (an AMPK antagonist) and siRNA downregulation of TXNIP and NLRP3 were also used to verify this result in vitro. Taken together, our studies strengthen the view that geniposide administration inhibits the pyroptosis mediated by NLRP3 inflammasome via the AMPK pathway to mitigate MI/RI, thus putting forward a new treatment strategy for cardiovascular diseases.

## Materials and methods

### Reagents and materials

Geniposide (≥ 98% pure) was obtained from Beijing Solarbio Science & Technology Co., Ltd. (Beijing, China). Cu/Zn/ Mn-SOD Assay Kit with WST-8 (#S0103) and the caspase-1 activity assay kit (C1102), The hematoxylin and eosin (H&E) staining kit (Cat#SBJ-1247) and RIPA lysis buffer (#P0013B) were purchased from Beyotime Institute of Biotechnology (Jiangsu, China). Antibodies toward the following proteins were used: β-actin and β-Tubulin from ZSGB-BIO (Beijing, China); p-AMPK(Th172) (#2535), AMPK (#2532), phospho-acetyl coenzyme A carboxylase (#11,818 p-ACC), acetyl coenzyme A carboxylase (#3662 ACC), Histone-3 (#4499) and TXNIP (#14,715) from Cell Signaling Technology (Danvers, MA, USA); N-GSDMD (ab215203), NLRP3 (#ab263899) and IL-1β (#ab9722) from Abcam (MA, USA); ASC (#bs-6741R) from Bioss (Beijing, China); cardiac troponin T (anti-cTnT, sc-8121) from Santa Cruz Biotechnology (Santa Cruz, CA, USA). Thioredoxin Polyclonal antibody(#14,999-1-AP) from Proteintech Group (Wuhan, China). AICAR (HY-13417) and Compound C (HY-13418A) were obtained from MCE (Shanghai, China). HRP-conjugated goat anti-mouse/rabbit secondary antibodies came from ZSGB-BIO (Beijing, China). PrimeScripttr RT reagent Kit with gDNA Eraser (# AK3801) and SYBR Premix Ex TaqTMII (# AKA303) were from Takara (Japan). IL-1β ELISA Kit (#EK0394) and IL-18 ELISA Kit (#EK0433) were purchased from Wuhan Boster Biological Technology Ltd. (Wuhan, China). Lipofectamine^™^2000 (Lip2000) was obtained from Invitrogen Life Technologies (Grand Island, NY, USA). 2,3,5-Triphenyl tetrazolium chloride and Evans Blue (Lot# BCCD5244) were purchased from Sigma-Aldrich (St. Louis, MO, USA).

### Animals

Male C57BL/6J (20–25 g) mice were ordered from Beijing Vital River Laboratory Animal Technology (Beijing, China). All experimental protocols were authorized by the Laboratory Animal Ethics Committee of Shantou Medical University (SUMC2019-412). Animals were handled under the International Guiding Principles for Biomedical Research Involving Animals (2012 version), issued by the Council for the International Organizations of Medical Sciences. The mice were housed in the experimental animal center of Shantou Medical University, which was maintained at temperature (25 ± 1 °C) and humidity (65 ± 5%) in 12:12 h light–dark cycle and provided with water ad libitum and standard diet. The animals were acclimatized for 7 days before experimentation.

### Myocardial ischemia/reperfusion injury (MI/RI) model

The left anterior descending (LAD) coronary artery of the mice was ligated temporarily, and then released it to make it unblocked according to a previously described method [29, 31]. In detail, we anesthetized the mouse using pentobarbital sodium at 40 mg/kg by intraperitoneal injection, and then occluded the LAD. To ensure the success of the surgery, an electrocardiogram was used to monitor the whole process continuously though the ST segment variation, which was changed by tightening or loosening the ligation. After 45 min of ischemia in mice, the coronary circulation was restored for 4 h to re-perfuse the myocardium. Mice were pretreated with intraperitoneal injection of GEN (10 or 20 mg/kg) 30 min before ischemia in the GEN group. The following four experimental groups were established: I/R + Vehicle (mice subjected to MI/RI and treated with vehicle); GEN 10 mg/kg group (mice subjected to MI/RI and treated with 10 mg/kg geniposide); GEN 20 mg/kg group (mice subjected to MI/RI and treated with 20 mg/kg geniposide), and the sham group (similar operations without LAD ligature). The dose was chosen according to previous study [32] and echocardiography was used to measure mouse cardiac functioning, and myocardial tissues and blood were collected and stored at −80 °C for testing of other indicators.

### Echocardiographic analysis

Echocardiographic analysis was detected according to a previously described method [33, 34]. After 24 h reperfusion, Mice were anesthetized by breathing in 1–2% isoflurane, allowing for noninvasive examination and put on a heating plate to maintain body temperature. Echocardiography equipment system (Visual Sonic Vevo 2100, Toronto, ON, Canada) used to assesse cardiac function by using a linear transducer. Left ventricular end-diastole diameter (LVEDd) and left ventricular end-systolic diameter (LVESd) were measured, respectively. M-mode echocardiograms were saved and used to detect cardiac function of mice. Fractional shortening (LVFS) and ejection fraction (LVEF) and were calculated using vevo LAB 3.1.0.

### TTC–Evans Blue and H&E staining

Infarct size after I/R injury was examinate as previously described [35, 36]. Briefly, after reperfusion 24 h, mice were anesthetized, the LAD artery was re-occluded at the previous ligation, 1 mL of 1% Evans blue (Sigma-Aldrich, St. Louis, MO, USA) was injected into the LV cavity and removed the mouse hearts rapidly by resection, washed twice with precooled phosphate-buffered saline (PBS, pH 7.4) to clear up the blood, and weighed. 2,3,5-triphenyltetrazolium chloride (TTC) staining was used to detect the myocardial infarct. The myocardium was uniformly cut into 4–5 pieces below the ligation site and then immersed in PBS containing 1% TTC incubation (37 °C, 15 min). The images of each group of stained sections were taken, and the planimetry method was used to measure the infarct area of each section by Image J software.

For H&E staining analysis, pre-chilled PBS buffer was used to wash away blood from the myocardium, then 4% paraformaldehyde fixation (4 °C, 24 h), then dehydrated in graded ethanol, cleared with xylene, and embedded in paraffin. Samples taken 2 mm below the ligature were then serially sectioned at 4 μm thickness and stained with H&E kit. Photograph the slides under a microscope (Zeiss Microsystems).

### TUNEL staining analysis

After 24 h of reperfusion, the heart was excised, and the myocardium was washed with pre-cooled PBS and then fixed in 4% paraformaldehyde at 4 °C for 24 h, then dehydrated in graded ethanol, cleared with xylene, and embedded in paraffin. The samples taken 2 mm below the ligation line were then serially sectioned at a thickness of 4 μm and performed using the DeadEnd^™^ Fluorometric TUNEL System (Promega, USA) according to the manufacturer’s protocols. DAPI solution (Beyotime, Shanghai, China) was used to label the cell nucleus. A Zeiss 800 confocal microscope (Zeiss Microsystems) was applied to observe the fluorescence.

### Hypoxia/reoxygenation model

Using a modification of a previously described protocol [37]. Neonatal rat ventricular myocytes (NRVMs) were isolated from whole hearts of neonatal 1–3 day old Sprague–Dawley rats. Briefly, hearts were minced and digested with 0.25% trypsin for 12 h, then terminate digestion with Dulbecco’s modified Eagle’s medium (DMEM) containing 10% fetal bovine serum (FBS), washed three times with PBS, and then digest with Type II collagenase (gibco, Thermo Fischer Scientific) at 37 °C. The digested cell suspension was pre-plated to clear fibroblasts. NRVMs were cultured in DMEM complete medium supplemented with 1% antibiotic–antimycotic mix and 100 μM bromodeoxyuridine (Sigma, St. Louis, MO, USA). Then after 3–4 days cells were subjected to H/R procedure as flowing group.

H9c2 cells were seeded at a density of 1 × 10^4^/cm^2^ in DMEM containing 10% FBS and antibiotics. Both H9c2 cells and NRVMs were washed with PBS, and 100% nitrogen was added to saturate the hypoxia buffer pH 6.2: 137 mM NaCl, 4 mM HEPES, 20 mM Na lactate, 12 mM KCl, 0.49 mM MgCl_2_ · 6H_2_O and 0.9 mM CaCl_2_ for 20–30 min. After saturation, cells were washed 2–3 times with hypoxic solution. Depending on the size of the petri dish, add different volumes of hypoxia buffer, and cells were incubated in a hypoxia workstation with 94% N_2_, 5% CO_2_, and 1% O_2_ for 3 h. Reoxygenation in normal glucose culture medium and incubation for another 2 h.

### CCK-8 analysis

The viability of NRVMs and H9c2 cells were evaluated by the CCK-8 Kit (Dojindo Laboratories, Kumamoto, Japan). Cells were seeded in 96-well plates, and 10 μl of detection solution was added to each well for detection and incubated for 2 h following the manufacturer’s instructions. Cell viability was calculated by the absorbance of 450 nm with a full-wavelength microarray (Thermo Fisher Scientific, Carlsbad, CA, USA).

### JC-1 examination

The chemical dye of 5,5′,6,6′-tetrachloro-1,1′,3,3′- tetraethylbenzimidazolcarbocyanine iodide (JC-1) (MedChemExpress, USA) was used to determine the mitochondrial membrane potential following the manufacturer’s protocol. Briefly, after H/R injury, we then incubated NRVMs with JC-1 at 37 °C for 20 min in a confocal 35-mm coverglass-bottom petri-dish. Before imaging, NRVMs were washed 2–3 times with PBS to remove residual dye. A Zeiss 800 confocal microscope (Zeiss Microsystems) was adopted to capture fluorescent images and analyze fluorescence intensity.

### Detection of serum myocardial enzymes

Mice were sacrificed after peripheral blood collection, serum was separated to detect myocardium damage enzymes, including lactic dehydrogenase (LDH), creatine kinase (CK) and creatine kinase-MB (CK-MB). The automatic chemistry analyzer (Toshiba Medical Systems Corporation, Tokyo, Japan) was used to measure these indicators. Cell supernatant LDH leakage was detected by LDH kits (Nanjing Jiancheng Bioengineering Institute, China) based on the manufacturer’s instructions. All test indicators were conducted in triplicate.

### Detection of serum inflammatory cytokines

ELISA kits were adopted to analyze the inflammatory factor of IL-1β and IL-18 in the serum performing as the manufacturer’s protocols. The absorbance of 450 nm was detected by a microplate spectrophotometer (Thermo Fisher Scientific, USA). Concentrations of the cytokines were calculated by reference to the standard curves.

### SOD2 activity

Mitochondria from mouse myocardium tissue and NRVMs were processed as group divided. According to the manufacturer’s protocols, SOD2 enzymatic activity was evaluated by a Cu/Zn-SOD and Mn-SOD Assay Kit with WST-8 (Beyotime, China), based on the capacity of SOD2 to competitively inhibit WST-8 by combining with superoxide radicals generated by xanthine oxidase. SOD1 inhibitors A and B were added to the sample to surpass the residual SOD1 activity, then samples were mixed with WST-8/enzyme working solution for 30 min at 37 °C. The absorbance of 450 nm was detected. When WST-8 formazan inhibition rate is 50%, SOD2 enzyme activity is defined as 1 unit. The protein concentration was analyzed by the BCA standard curve.

### RNA interference

The siRNAs of TXNIP, NLRP3, and control scramble siRNA were ordered from Biotend Co.,Ltd. (Shanghai, China), The sequence of targeting siRNA for rat TXNIP was 5′-CAUCCUUCGAGUUGAAUAUTT-3′, siRNA for rat NLRP3 was 5′-CCUGUCUUUGCCGTAGAUUACCGUAAG-3′. Following the manufacturer’s protocols, one day before transfection, 3 × 10^5^ H9c2 cells were seeded on a 6-well plate with 2.0 ml of DMEM cell culture medium containing FBS and antibiotics, estimating that cells should reach 70–90% confluence within 24 h. For transfection, 50 μM siRNA was added to 100 μl of serum-free DMEM in an eppendorf tube and mixed gently; in another tube, 3 μl Lip2000 was diluted into 100 μl serum-free DMEM and incubate for 5 min at room temperature, then the diluted siRNA and Lip2000 reagent were mixed; the normal medium was change to serum-free DMEM, and the transfection mixture were added. After the cells were incubated for 4–6 h at 37 °C, the transfection complexes were removed and replaced with fresh medium. After transfection, H9c2 cells were culturing at 37 °C for 24 h–48 h, then RT-PCR and western blotting verified the knockdown efficiency and following the other experiments.

### Western blotting and immunoprecipitation

The total protein of cells and myocardium tissues were extracted by RIPA lysis solution containing 1% PMSF. The BCA kit (Thermo Fisher Scientific Inc., Rockford, IL, USA) was adopted to measure the protein concentration of sample. Equal quantities of protein were separated by 8–12% SDS-PAGE and transferred onto nitrocellulose membranes. Membranes were blocked with 5% skimmed milk in PBST buffer (PBS containing 0.2% Tween 20) at room temperature for 1 h and then immunoblotted with primary antibodies: p-AMPK (1:1000), AMPK (1:1000), p-ACC (1:1000), ACC (1:1000), NLRP3 (1:2000), ASC (1:1000), N-GSDMD (1:1000), Cleaved caspase-1 (1:1000), IL-1β (1:1000), TXNIP (1:1000), β-actin (1:2000), β-Tublin (1:2000) and Histone-3 (1:3000) overnight at 4 °C. The membranes were washed and incubated with HRP-conjugated secondary antibodies for 1 h at room temperature. Signals were detected by a SuperSignal detection kit (Thermo Fisher Scientific, USA).

For immunoprecipitation, before H/R manipulation, NRVMs were pre-treated with 40 μM GEN for 0.5 h. After that, the cells were washed two times with PBS and lysed on ice for 15 min, the cell lysates were centrifuged at 12,000*g* for 20 min and then the supernatant was collected. Next, anti-TXNIP, anti-NLRP3 and anti-thioredoxin Ab was used to immunoprecipitated overnight at 4 °C and then precipitated with protein A + G agarose beads (Beyotime, China) for 2 h. And then the protein A + G agarose beads were washed 4 times with the lysis buffer. The beads were boiled with 1% SDS loading buffer for western blotting with the specified antibodies.

### Immunofluorescence and nuclear/cytoplasmic fractionation.

NRVMs were seed at glass cover slips in a dish. After exposure to different treatment, the cell was washed with PBS for 2–3 times, fixed with 4% paraformaldehyde for 20 min and then permeabilized with 0.5% Triton X‐100 at 25 °C for 30 min. Next, the cells were incubated with specific primary antibody against p-AMPK (1:200), TXNIP (1:200), NLRP3 (1:200) overnight at 4 °C. The cells were washed three times with PBS and subsequently incubated with a fluorochome‐labelled secondary antibody for 1 h at room temperature. The cells were then washed three times with PBS and the nuclei were counterstained with DAPI for 5 min at room temperature. A Zeiss 800 confocal microscope (Zeiss Microsystems) was used for imaging.

Based on manufacturer’s protocols, NE-PER nuclear and cytoplasmic extraction reagents (Thermo Fischer Scientific, USA) were adopted to extract cytoplasmic and nuclear protein of cultured cells.

### Quantitative RT-PCR

Total RNA was separated and extracted by Trizol (TaKaRa Biotechnology, Japan). RNA concentration quantities by nanodrop2000 (Thermo Fisher Scientific, Carlsbad, CA, USA). PrimerScript^®^ RT reagent Kits with gDNA Eraser Kits (TaKaRa Biotechnology, Japan) were used to reverse 1 μg total RNA transcribed into cDNA. RT-PCR experiment was performed by SYBR^®^ Premix ExTaq^™^ II kits (TaKaRa Biotechnology, Japan) on an ABI LIFE QuantStudio 12K detection system (Applied Biosystems, Foster City, CA, USA). Total reaction volume was 10 μl contained 1 μl cDNA in a template. The primers used are listed in Table 1 and the relative mRNA expression in each group was detected by using the comparative Ct (2^−ΔΔCT^) method in reference to GAPDH. A melting curve of each amplicon was determined to verify its specificity.

**Table 1.** Primers used for real-time PCR in this study

Genes	Forward Primer Sequence	Reverse Primer sequence
NLRP3 (mouse)	5′-AGAAGAGACCACGGCAGAAG-3′	5′-CCTTGGACCAGGTTCAGTGT-3′
ASC (mouse)	5′-ACTTCTGTGACCCTGGCAATGAG-3′	5′-GCTGAGCAGCTGCAAACGAC-3′
IL-1β (mouse)	5′-GAAATGCCACCTTTTGACAGTG-3′	5′-TGGATGCTCTCATCAGGACAG-3′
IL-18 (mouse)	5′-GCCATGTCAGAAGACTCTTGCGTC-3′	5′-GTACAGTGAAGTCGGCCAAAGTTGTC-3′
GAPDH (mouse)	5′-TGACCTCAACTACATGGTCTACA-3′	5′-CTTCCCATTCTCGGCCTTG-3′
NLRP3 (rat)	5′-TCTGTTCATTGGCTGCGGAT-3′	5′-TAGCCGCAAAGAACTCCTGG-3′
TXNIP (rat)	5′-TACAGGTGAGAACGAGATGGTGA-3′	5′-TTGAGTTGGCTGGCTGGGAC-3′
GAPDH (rat)	5′-AACGACCCCTTCATTGACCTC-3′	5′-CGCCAGTAGACTCCACGACATA-3′

### Statistical analysis

All data presentations are shown as mean ± standard deviation (SD) unless otherwise stated. Significance between two groups was performed by Student’s two-tailed t-test with GraphPad Prism 6.01 (GraphPad Software, La Jolla, CA, USA). In other cases, significance for more than two groups was done using one-way ANOVA in Prism. Differences were considered significant at P < 0.05.

## Results

### Geniposide ameliorated cardiac damage and improved cardiac function of the mice suffering from myocardial ischemia/reperfusion injury (MI/RI)

In our study, the total number of experimental animals consumed in the experiment (Additional file 1: Figure S1 D,G)and the number of mice in each group were shown(Additional file 1: Figure S1 A, B, C, F). At the same time, our MI/R surgery didn't affect the survival rate of mice in each group, as shown the chi-square test between the groups(Additional file 1: Figure S1 E,H).Subsequently, echocardiography was used to evaluate changes in cardiac function and structure. Cardiac functions were determined 24 h after MI/RI operation. Compared with the sham group, the MI/RI mice had significantly reduced left ventricular ejection fraction (LVEF) and left ventricular fractional shortening (LVFS) and increased left ventricular end-diastole diameter (LVEDd) and left ventricular end-systolic diameter (LVESd). However, these changes were drastically reversed by geniposide (Fig. 1C). These data indicated that geniposide improved cardiac function in MI/RI mice. Moreover, we evaluated the myocardial injury by examining the changes of myocardial damage biomarkers in the serum. The levels of serum LDH (Fig. 1D), CK (Fig. 1E), and CK-MB (Fig. 1F) in the MIRI mice were significantly increased, while a notable decrease was found in the MI/RI + GEN 20 mg/kg and MI/RI + GEN 10 mg/kg treated mice (Fig. 1D–F).

To investigate the protective effects against MI/RI in mouse model, TTC staining was adopted to determine the size of myocardial infarction after reperfusion for 24 h. The result showed that infract size rose 34% compared with sham group, and geniposide restored infarct size to 22% over sham (Fig. 1G). I/R-induced pathological was performed to further evaluate the oxidative reaction. DHE staining was performed in myocardial tissue with MI/RI as a marker of free radical formation. As a result, obviously increased infract size after MI/RI was relieved by geniposide.

The pathological changes of mice cardiac structure were detected by H&E staining. The results suggested that the morphology of myocardial cells in the MI/RI group was deformed, and the edges of the cells were blurred compared with sham group. Geniposide improved myocardial damage in MI/RI group. The results indicated that geniposide can improve MI/RI damage (Fig. 1H).

### Geniposide induced AMPK activation and alleviated oxidative stress level

**Fig. 2:**
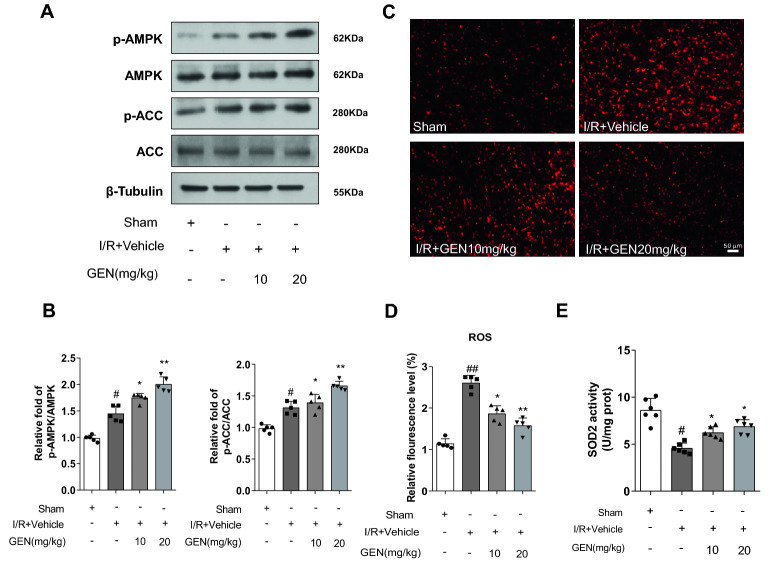
Geniposide alleviates cardiac damage caused by MI/R injury. **A** The protein expression of p-AMPK, AMPK, p-ACC, and ACC in mouse myocardium presented by representative western blot bands (n = 5). **B** The protein expression of p-AMPK and p-ACC in mice cardiac tissues of each group were shown by statistical histograms of representative western blots. **C** The representative DHE staining of the left ventricular of the mice, scale bar = 100 μm (n = 6). **D** Quantitative analyses of DHE staining level. **E** SOD2 activity of mouse heart samples were assayed using a commercial kit (n = 6). The data for each group were shown as the mean ± SD; ^#^p < 0.05, ^##^p < 0.01 vs. Sham group; *p < 0.05, **p < 0.01 vs. I/R + Vehicle group

As Fig. 1C shows, geniposide can improve the cardiac function after MI/RI in mice. The mouse heart requires a huge amount of energy to maintain normal physiological cardiac function. The body’s energy metabolism is closely associated with the cardiac function. To investigate the specific mechanism by which geniposide improves MI/RI, we tested the level of energy-related proteins. The expression of phosphorylated AMPKα (p-AMPK) and phosphorylated acetyl-CoA carboxylase (p-ACC), a substrate of AMPKα, was apparently increased in the I/R group than that of the sham group (Fig. 2A, B), and geniposide upregulated the protein expression of p-AMPK and p-ACC in the I/R + GEN group (Fig. 2A, B). Our results indicated that geniposide may decrease myocardial damage in MI/RI by promoting energy metabolism related proteins.

Moreover, DHE staining fluorescence intensity and SOD2 activity in tissue were apparently decreased in mice of I/R + Vehicle group compared to the sham group. Mice treated with GEN 20 mg/kg showed a weaken fluorescence intensity of DHE staining (Fig. 2C, D), while SOD2 activity (Fig. 2E) was elevated compared with the I/R + Vehicle group. In addition, 10 mg/kg GEN treatment was consistent with 20 mg/kg GEN treatment.

### *Geniposide treatment inhibited NLRP3 inflammasome*-*mediated pyroptosis in the cardiac tissues of MI/RI*

**Fig. 3:**
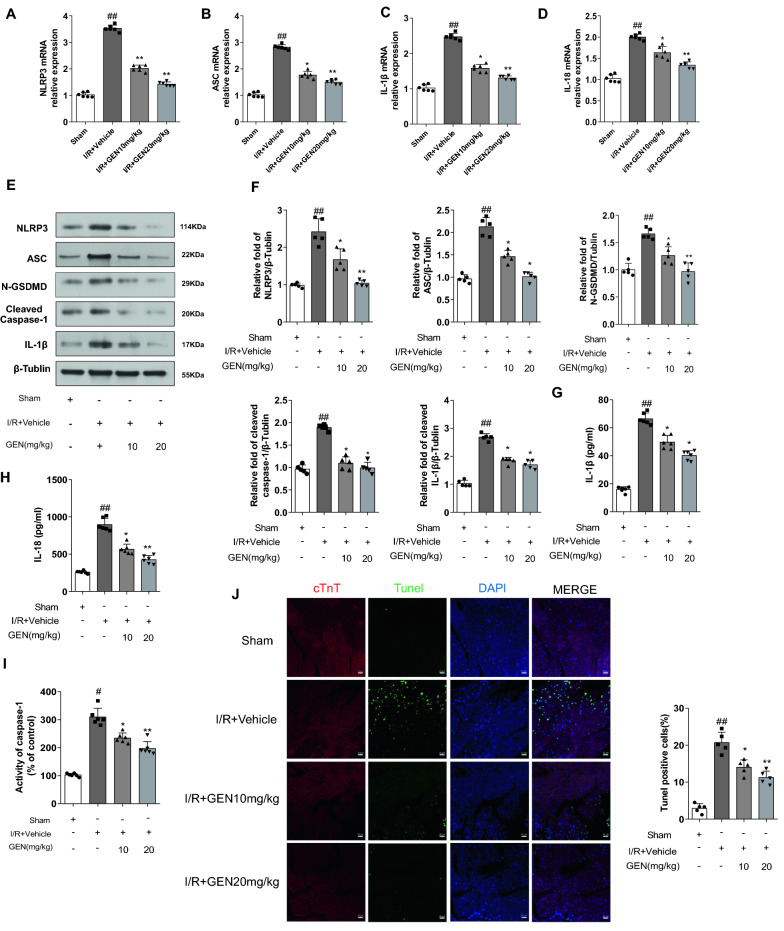
GEN exerts its protective effect by activating AMPKα and suppressing NLRP3 inflammasome activity. **A**–**D** The mRNA expression of NLRP3, ASC, IL-1β, and IL-18 in mice myocardium of each group detected by RT-PCR (n = 5). **E** Representative western blotting results of NLRP3, ASC, N-GSDMD, cleaved caspase-1, and IL-1β (n = 6). **F** Densitometry data for NLRP3, ASC, N-GSDMD, cleaved caspase-1, and IL-1β in myocardium of each group of mice. **G**,** H** Inflammatory factor of IL-1β and IL-18 in the serum after GEN treatment (n = 5). **I** Myocardial caspase-1 activity was assessed in each group (n = 5). **J** Immunofluorescence TUNEL staining after GEN treatment (n = 6); scale bar = 20 μm. The data for each group were shown as the mean ± SD; ^#^p < 0.05, ^##^p < 0.01 vs. Sham group; *p < 0.05, **p < 0.01 vs. I/R + Vehicle group

ELISA results from the mice with MI/RI indicated that the level of IL-18 and IL-1β increased dramatically, which suggested the outbreak of inflammation in the myocardium following MI/RI insult. The results shown MI/RI + GEN 10 mg/kg group exhibited low expression levels of myocardial IL-18 and IL-1β. The MI/RI + GEN 20 mg/kg group also showed a significant decreased in IL-18 and IL-1β levels (Fig. 3H, I). Moreover, Western blotting result showed that compared with the sham group mice, MI/RI mice apparently upregulated the expression of NLRP3 along with cleaved caspase-1 and IL-1β, that are the downstream factors of the active NLRP3 inflammasome. These results suggested that activated NLRP3 inflammatory bodies play an important role in the process of MI/RI. In contrast, the MI/RI + GEN 20 mg/kg group exhibited a decrease expression in myocardial NLRP3 (Fig. 3E).

We then investigated whether geniposide regulates NLRP3 downstream signals by suppressing the expression of NLRP3. The results of TUNEL assays suggested that the level of apoptosis was notably elevated by MI/RI, indicating that there was either pyroptotic cell death or apoptotic occurring in these cardiomyocytes caused by MI/RI (Fig. 3J). According to this result and the previous studies of the NLRP3 inflammasome activation [6], we hypothesize that pyroptosis is involved in MI/RI development in mice with MI/RI. We detected the activation of two key proteins, N-GSDMD and cleaved caspase-1 in pyoptosis cell death. The results of western blotting suggested that the levels of cleaved caspase-1 and N-GSDMD significantly increased in the mice with MI/RI (Fig. 3E). In addition, the higher level of caspase-1 activity and cleaved caspase-1 were elevated in the myocardia of MI/RI mice (Fig. 3I). In short, the above results indicated that pyroptosis was widespread in the mice with MI/RI, and treatment with 20 mg/kg geniposide significantly inhibited the pyroptosis mediated by inflammatory bodies during MI/RI, as shown by the marked decrease of N-GSDMD and cleaved caspase-1 expression as well as the level of caspase-1 activity and percentage of TUNEL-stained cardiomyocytes (Fig. 3E, F, J). Similarly, treatment with 10 mg/kg geniposide decreased the expression level of N-GSDMD, cleaved caspase-1 and IL-1β (Fig. 3E, F).

Additionally, RT-PCR results shown that the transcription level of NLRP3, N-GSDMD, ASC, and IL-1β mRNA were notably upregulated in the myocardium tissues of MI/RI mice compared with those of sham, and all doses of GEN significantly suppressed the mRNA level of NLRP3, ASC, IL-1β, and IL-18 (Fig. 3A–D). Together, our result indicate that GEN treatments decreased pyroptosis in MI/RI mice.

### Geniposide improved the cell damage caused by H/R in NRVMs

**Fig. 4:**
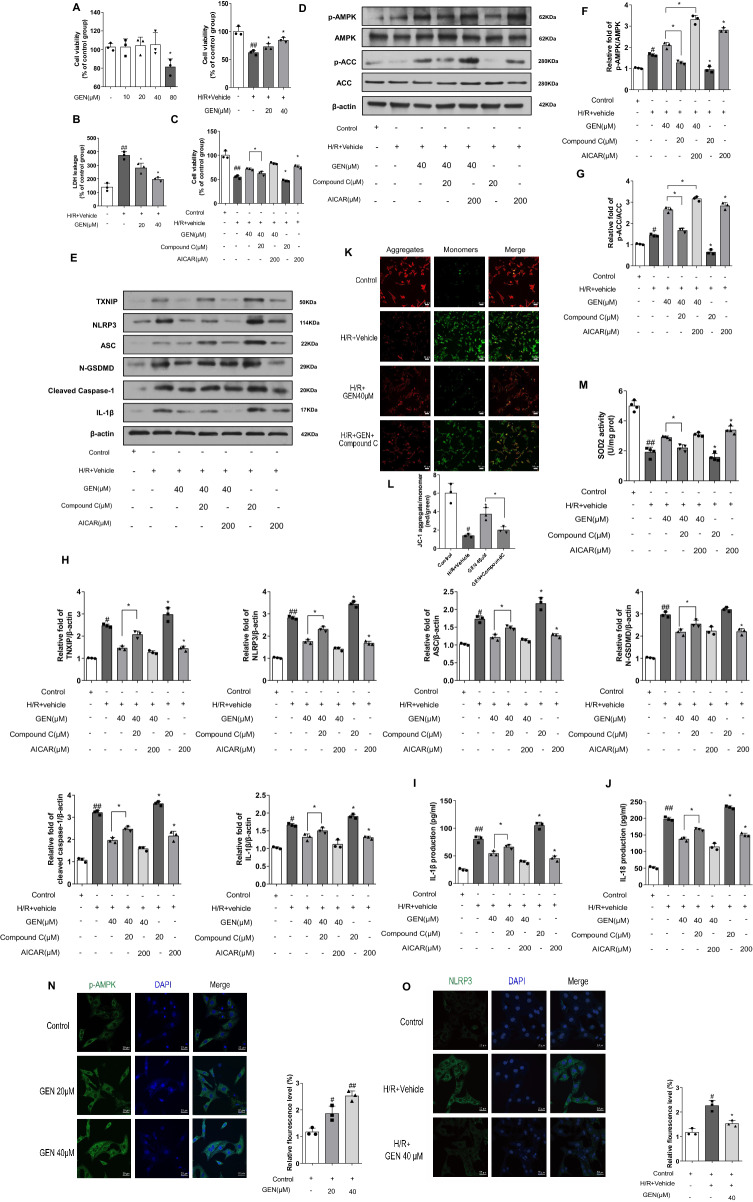
GEN ameliorates the cell injuries caused by H/R in NRVMs. **A** CCK-8 assay detected cell viability of NRVMs (n = 3). The results are presented as a percentage of the control. Cell viability of NRVMs following different concentrations of GEN exposure was measured by CCK-8 assay. NRVMs were treated with 20 μM and 40 μM with or without H/R insult. **B**,** C** Cell supernatants of LDH leakage and the change of cell viability in NRVMs. The cells were treated with the indicated treatments. **D**,** E** Expression of p-AMPK, AMPK, p-ACC, ACC, TXNIP, NLRP3, ASC, cleaved caspase-1, N-GSDMD-, IL-1β proteins detected by western blotting (n = 3). **F**,** H** Densitometry data of p-AMPK, p-ACC, ACC, TXNIP, NLRP3, ASC, cleaved caspase-1, N-GSDMD-, IL-1β in myocardium of each group of mice. **I**,** J** IL-1β and IL-18 in cell supernatants were detected by ELISA assays. **K**,** L** Intracellular red and green fluorescence of JC-1 was determined using a confocal microscope. The aggregates and monomers were assessed (n = 3); scale bar = 50 μm. **M** SOD2 enzymatic activity of NRVMs assayed with a commercial kit. **N** Representative images of immunofluorescence staining of p-AMPK in cardiomyocytes after different doses of GEN treated and the calculated intensity of p-AMPK (n = 3); scale bar = 20 μm. **O** Representative images of immunofluorescence staining of NLRP3 after H/R insult with 40 μM GEN treatment and the calculated intensity of NLRP3 (n = 3); scale bar = 20 μm. The data for each group were shown as the mean ± SD; ^#^p < 0.05, ^##^p < 0.01 vs. Control group; *p < 0.05, **p < 0.01 vs. H/R + Vehicle group

To further identify the cellular cardioprotective function of geniposide, we modeled in vitro H/R in NRVMs. NRVMs treated with escalating doses of geniposide showed viability that was not notably different from control treated with PBS until reaching 80 μM genoposide concentration (Fig. 4A). Geniposide also blocked H/R injury in a dose-dependent manner, which was featured by LDH reduction, a vital indicator of the cell membrane integrity, and the increase of cell viability. Specifically, the cell viability of the H/R + Vehicle group was lower than that of the normal group while the LDH leakage had increased. The treatment with geniposide potently ameliorated the reduction in H/R-induced cell viability and decreased LDH leakage (Fig. 4A, B). Our results indicated that geniposide ameliorated the cell injuries stimulated by H/R.

Given the previous reports that AMPKα activity is beneficial towards cardiovascular diseases, we further proposed the hypothesis that the cardioprotective of geniposide against H/R injury was mediated by the activation of AMPKα. To explore the potential regulatory mechanism of AMPKα activation, we first detected the activation of p-AMPK in NRVMs. GEN at 40 μM was applied to NRVMs for hypoxia 3 h and reoxygenation for 2 h, and AMPK and p-AMPK levels were determined by western blot. As a result, p-AMPK expression upregulated in response to 40 μM geniposide treatment after H/R (Fig. 4D). Moreover, immunofluorescence results show pretreatment with geniposide for 4 h dose-dependently activated the expression of p-AMPK in normal condition (Fig. 4N). Consistently, transactivation of AMPK downstream expression of p-ACC was also significantly induced (Fig. 4D). These results suggest that the damage of NRVMs resulting from the H/R injury was significantly improved in the NRVMs treated with geniposide and the mechanism involved activities of energy metabolism.

### *Geniposide suppressed NLRP3 inflammasome in an AMPK-dependent manner *in vitro

Based on activation of AMPKα in H/R injury by GEN 40 μM, we incubated NRVMs with the AMPK antagonist Compound C. In addition, the effect of the combination of AMPK agonist AICAR and geniposide on H/R injury was also confirmed by CCK-8 assay. Considering the results that geniposide alleviates H/R injury in vitro and AMPK was also related to H/R-induced cardiac cell injury, geniposide protected against H/R injury in a dose-dependent way. In contrast, co-culture of Compound C with geniposide completely blocked these protective actions (Fig. 4C). Moreover, to determine whether geniposide attenuates H/R-induced mitochondrial dysfunction, MMP levels were increased. JC-1 is a sensitive fluorescent dye that forms monomers in damaged and depolarized mitochondria and form aggregates in normally polarized mitochondria. With the depolarization of the mitochondrial membrane, the color of this dual-emission probe changes from red orange to green. As shown in Fig. 4K**,** the cells of the control group are clearly red, whereas the H/R damage quickly leads to the dissipation of MMP, as illustrated in the increased intensity of green fluorescence and the concomitant disappearance of red fluorescence. Geniposide pretreatment notably reduced the change of MMP, which manifested that the green fluorescence was suppressed, and the red fluorescence recovered. Compound C abolishes the protective effect of geniposide. Similarly, SOD2 activity showed consistent results with JC-1(Fig. 4M).

### *Pyroptosis suppression is crucial regulation for the protective of geniposide *in vitro

Recently, the GSDMD has been described as a novel type of pro-inflammatory programmed cell death initiated by activated inflammasome and processed by caspase-1, and has been proved to contribute to cardiomyocyte damage induced by H/R. In vivo, we have demonstrated GEN inhibits the expression of NLRP3 after MI/RI. Similarly, after H/R insult, GEN 40 μM treatment effectively inhibited the expression of NLRP3 (Fig. 4E, O), ASC, N-GSDMD, cleaved caspase-1, and IL-1β (Fig. 4E, H). Compound C suppressed the function of geniposide on the protein expression of TXNIP, NLRP3, ASC, caspase-1 and N-GSDMD (Fig. 4E, H). Moreover, cell viability in the model group was apparently decreased compared with the normal group and GEN 40 μM alleviated H/R damage. After treatment with Compound C, the viability of cells was lower than that in H/R + GEN 40 μM (Fig. 4C), while AICAR has a similar result on the expression of TXNIP, NLRP3, ASC, caspase-1 and N-GSDMD (Fig. 4E, H). Similarly, IL-1β and IL-18 in cell supernatants were determined by ELISA assays shown the consistent results (Fig. 4I, J). All in all, these results indicated that the geniposide effect on pyroptosis was reversed by Compound C.

### *Compound C counteracted the protective effects of geniposide *in vivo

**Fig. 5:**
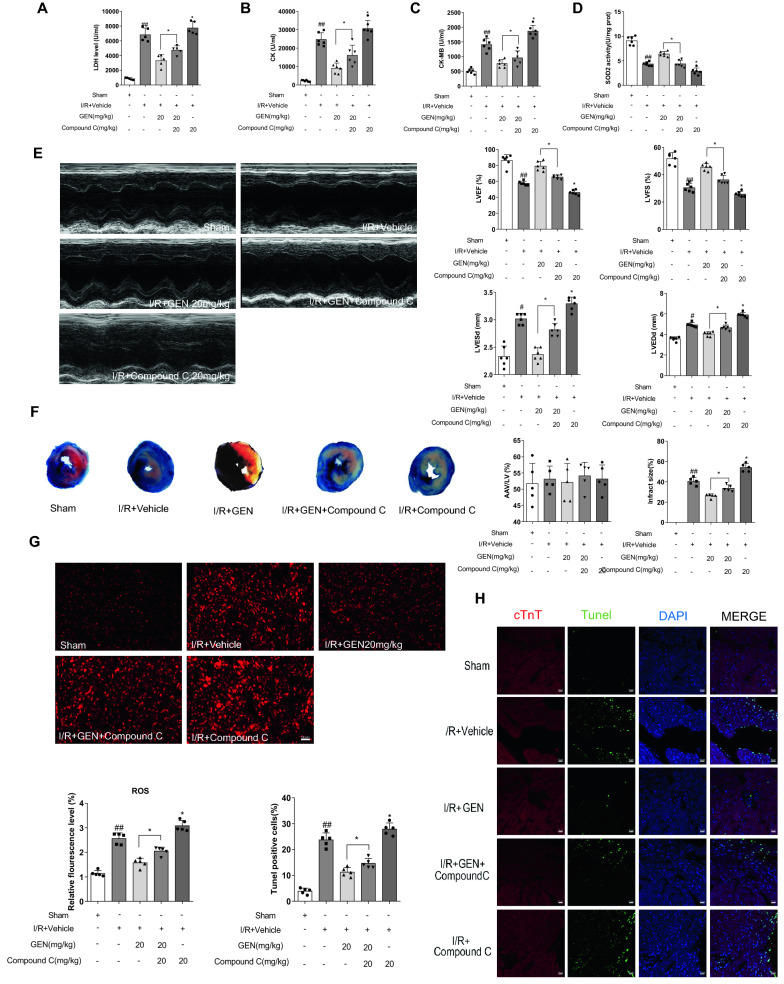
Compound C counteracts the cardioprotective effects of geniposide in mice. **A**–**C** Quantification of myocardium tissue (LDH, CK, CK-MB) on plasma of MI/RI mice treated with Compound C or GEN (n = 6). **D** SOD2 enzymatic activity of mice as indicated group assayed with a commercial kit (n = 5). **E** Quantitative assessment of analysis of LV ejection fraction (LVEF), fractional shortening (LVFS), left ventricular end-diastole diameter (LVEDd), and LV end-systolic diameter (LVESd) (n = 6). **F** Representative TTC–Evans Blue stained sections of hearts and quantitative data of the LV infarct size. Infarct size (%) was expressed as the percentage of infarct area relative to the total left ventricular area. the nonischemic section shown in blue area, red represent risk area, and the infarct region is stained white (n = 6). **G** Detection of ROS in myocardium by DHE staining. Compared with sham, the DHE fluorescence intensity in the myocardium of the vehicle-treated infarcted group was sharply increased and Compound C ablated the protective effect of geniposide (n = 6). **H** Immunofluorescence TUNEL staining after Compound C or GEN treatment (n = 6). The data for each group were shown as the mean ± SD; ^#^p < 0.05, ^##^p < 0.01 vs. Sham group; *p < 0.05, **p < 0.01 vs. I/R + Vehicle group

Subsequently, we further explored whether mice treated with Compound C after geniposide treatment demonstrated reversal of the morphological changes in vivo. Our result suggest that mice treated with additional Compound C shown an exacerbated MI/RI which was characterized by increased LDH, CK, CK-MB (Fig. 5A–C) and SOD2 activity (Fig. 5D). Similarly, DHE staining results showed the activated AMPKα and the protective effect against oxidative stress produced by geniposide were eliminated by Compound C (Fig. 5G). Moreover, echocardiography showed that compared with the sham group, the I/R + Vehicle group had a significant LVEF and LVFS decrease and LVEDd and LVESd increase in MI/RI mice. Cardiac function was improved by geniposide in the I/R + Vehicle group, and Compound C reversed the protection of geniposide (Fig. 5E).

Evans blue and triphenyltetrazolium chloride staining were performed on cardiac sections after reperfusion for 24 h. As shown in Fig. 5F. The non-hazardous Evans blue perfusion area is stained blue, while the viable myocardium is stained red, and the infarcted myocardium appears pale. Compared to mice in the GEN 20 mg/kg group, GEN 20 mg/kg + Compound C mice have a larger infract size. Mice treated with Compound C alone exhibited aggravated injury compared to the I/R + Vehicle group (Fig. 5F). Furthermore, the TUNEL assay indicated that the cell apoptotic rate of myocardium tissue was markedly elevated after MI/RI insult. Geniposide at 20 mg/kg suppressed the apoptosis of myocardium. Compound C abolished the protective effect produced by geniposide for MI/RI (Fig. 5H).

### Geniposide inhibited the TXNIP/NLRP3 complex formation in NRVMs

**Fig. 6:**
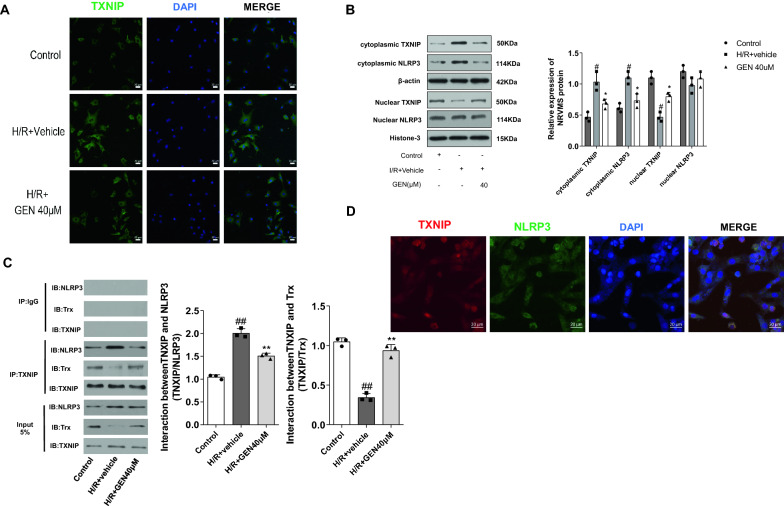
Geniposide inhibits the activation of the TXNIP/NLRP3 complex formation in NRVMs. **A**, **B** Translocation of TXNIP occurs in NRVMs. Immunofluorescence staining for TXNIP, where DAPI is used to locate the nuclei of the cells, and Western blotting of TXNIP in the 3 groups (n = 3). TXNIP located in nucleus in control group and shifted to cytoplasm in H/R + Vehicle group, and geniposide at 40 μM reversed the TXNIP localization from the cytoplasm to nucleus. **C** After reoxygenation, the interaction of TXNIP/NLRP3 was determined by coimmunoprecipitation (Co-IP) in the NRVMS of the 3 groups. Cardiomyocytes samples of the H/R + Vehicle and GEN 40 μM groups were harvested after reoxygenation (n = 3). **D** Quantitative analysis of TXNIP/NLRP3. The data for each group were shown as the mean ± SD; ^#^p < 0.05, ^##^p < 0.01 vs. Control group; *p < 0.05, **p < 0.01 vs. H/R + Vehicle group

In our research, cardiomyocytes level of NLRP3, apoptosis-associated speck-like protein (ASC), caspase-1, as well as IL-1β and IL-18, were increased in the H/R + Vehicle group compared with the control group. Moreover, geniposide apparently decreased the expression of the proteins above (Fig. 4E). We then explored whether geniposide suppressed the NLRP3 inflammatory bodies by regulating TXNIP. Our findings suggested that the expression of TXNIP was notably higher in H/R + Vehicle group than that in the control group (Fig. 4E). These results demonstrated that the protein expression of TXNIP and its functional status were participated in the process of activating NLRP3 inflammasome. Furthermore, coimmunoprecipitation was conducted that proved the formation of the TXNIP/NLRP3 complex was increased in the MI/RI group compared with the control group (Fig. 6C). Consistently, confocal scanning further confirmed that TXNIP and NLRP3 colocalized in NRVMs (Fig. 6D).

Compared with the H/R + Vehicle group, geniposide treatment decreased TXNIP/NLRP3 complex formation. Meanwhile, the combination of TXNIP/Trx complex was decreased in the H/R + Vehicle group than that in control group; nevertheless, geniposide maintained the mutual interaction (Fig. 6C). In addition, we further confirmed that the ROS level, which was regarded as a key factor in activating the NLRP3 inflammasome, was improved by geniposide. Thus, these data suggest that geniposide treatment suppressed the binding of the TXNIP/NLRP3 complex followed by NLRP3 inflammasome activation.

We speculated that cellular localization of TXNIP may relate with the activation of NLRP3 inflammasome during MI/RI. Therefore, we examined their subcellular, specifically nuclear and cytoplasmic, localization. Our results showed that TXNIP was mainly localized in the nucleus in the control group. Moreover, western blotting and immunofluorescence results indicated that TXNIP translocated from the nucleus into the cytoplasm in the H/R + Vehicle group, whereas geniposide reversed this phenomenon (Fig. 6A, B). Moreover, immunofluorescence staining demonstrated that NLRP3 was also generated in the nucleus region in control group and there was a slight decrease in the nuclear region of the H/R + Vehicle and GEN 40 μM groups. Meanwhile, we could not observe NLRP3 in the cytoplasm of the control group by IF staining, but it exhibited an apparent increase in the cytoplasm of the H/R + Vehicle group; GEN 40 μM reversed this increase (Fig. 4M). Western blot results suggested that there was no remarkable change for expression of NLRP3 in the nucleus among these groups while the expression of NLRP3 in cytoplasm was increased in the H/R + Vehicle group and geniposide suppressed this expression (Fig. 6B).

### H/R injury of H9c2 cells was ameliorated by knockdown the expression of TXNIP and NLRP3

**Fig. 7:**
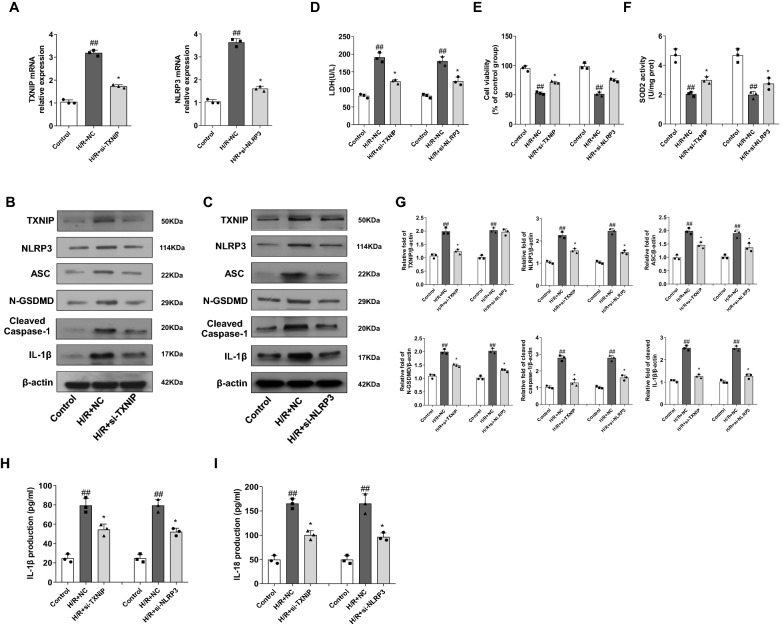
Effect of TXNIP and NLRP3 siRNA on inflammasome of H/R stimulated H9c2 cells. After application of negative control siRNA (NC siRNA) or TXNIP and NLRP3 siRNA for 48 h, H9c2 cells were exposed to hypoxia for another 3 h, and subsequently reoxygenation for 2 h. **A**–**C** Protein expression of TXNIP and NLRP3 was detected by Western blot at 48 h post-transfection, mRNA levels of TXNIP and NLRP3 in H9c2s were detected by RT-PCR at 24 h post-transfection and protein expression levels of ASC, N-GSDMD, cleaved Caspase-1 and IL-1β were analyzed by western blot. **D**,** E** Cell viability and LDH leakage after GEN treatment or using si-TXNIP or siNLRP3 (n = 3). **F** SOD2 enzymatic activity of H9c2 cells for the indicated groups, assayed using a commercial kit(n = 3). **G** IL-1β and IL-18 in cell supernatants were determined by ELISA assays. The data are presented as mean ± SD from three independent experiments. The data for each group were shown as the mean ± SD; ^#^p < 0.05, ^##^p < 0.01 vs. Control group; *p < 0.05, **p < 0.01 vs. H/R + Vehicle group

**Fig. 8:**
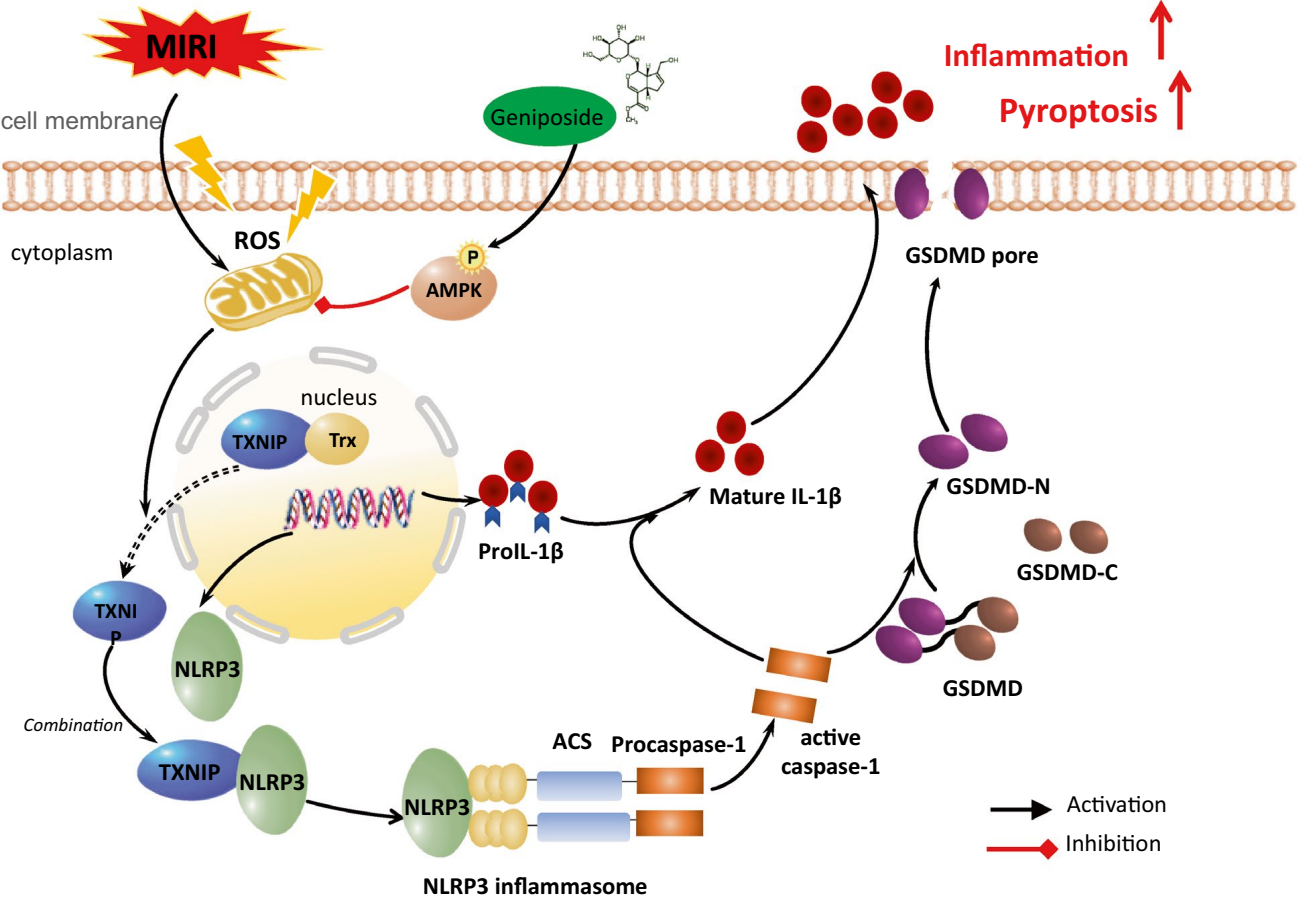
The underlying molecular mechanisms of the protective effects of geniposide against the myocardial ischemia/reperfusion injury of mice. Scheme summarizing the protective effects of GEN on MI/RI via activating of AMPK signaling and inhibiting of ROS/TXNIP/NLRP3 mediating inflammation and subsequent pyroptosis-related signaling pathways. Under physiological conditions, Trx binds to TXNIP in its reduced form and suppresses activity. However, MI/RI amplifies ROS overproduction, which results in Trx dissociating from TXNIP, and thereby TXNIP/NLRP3 activation. This process leads to inflammation and pyroptosis. Importantly, GEN upregulates the AMPK signaling pathway, contributing to inhibit MI/RI-induced oxidative stress and TXNIP/NLRP3 activation, which were effectively inhibition inflammation and subsequent pyroptosis

Based on the results that NLRP3 expression increased, and NLRP3 directly combines with TXNIP during MI/RI, we inferred that there was a deeper relationship among NLRP3, TXNIP, and MI/RI. H9c2 cells were transfected with si-NLRP3 or si-TXNIP, and after 48 h they were treated with H/R insult. RT-PCR and western blots analysis confirm that NLRP3 and TXNIP at the mRNA and protein level were significantly decreased (Fig. 7A–C). Meanwhile, CCK-8 assay result reveled that the viability of H9c2 cells treated by H/R was significantly increased after TXNIP or NLRP3 siRNA transfection and LDH leakage was decreased. Treatment with NLRP3 or TXNIP siRNA showed the better cell function (Fig. 7D, E). Similarly, SOD2 activity decreased after H/R insult, while si-TXNIP or si-NLRP3 transfection significantly suppressed the oxidative stress of H9c2 cells (Fig. 7F). Moreover, knockdown of TXNIP or NLRP3 expression significantly inhibited the expression of ASC, N-GSDMD, cleaved caspase-1, and IL-1β (Fig. 7B, C), and ELISA results were in agreement (Fig. 7H, I). These results indicated that H9c2 cells pyroptosis decreased and reduced MI/RI by suppressing TXNIP and NLRP3 expression (Fig. 8).

## Discussion

In the present study, we evaluated the potential mechanism of geniposide cardioprotection though AMPK/TXNIP/NLRP3 signaling pathway in NRVMs, H9c2 cells, and mice. Previous basic research has shown that geniposide provides a protection against MI/RI [38]. Here, the findings revealed that geniposide exerts a protective effect on MI/RI by the AMPK/TXNIP/NLRP3 signaling pathway. Moreover, we found that the decrease of NLRP3, ASC, N-GSDMD, cleaved caspase-1 and IL-1β proteins considerably repressed the activation of the NLRP3 inflammasome and reduced the production of downstream proinflammatory mediators in myocardial tissue, including IL-18 and IL-1β and subsequent cardiomyocyte pyroptosis, thereby mitigating MI/RI. However, this cardioprotective effect is weakened or dissipated under compound C or knockdown of NLRP3 or TXNIP expression. Under the treatment of AICAR, an agonist of AMPK, myocardial protection can be restored, signifying that the AMPKα signaling pathway is an important mechanism that mediates the beneficial function of geniposide (Fig. 8).

Myocardial injury is usually accompanied by cardiomyocyte apoptosis [39]. As a cytoplasmic receptor, NLRP3 responds to a series of molecules related to cellular ROS level and excessive production of pro-inflammatory cytokines [40, 41], and mediates caspase-1-dependent programmed cell death, called pyroptosis, that plays an important role in cardiovascular disease. Due to external stimuli, such as hypoxia and ischemia, NLRP3 activates caspase-1, which promotes the conversion of IL-1β and IL-18 precursor proteins to their respective active forms [13]. We detected the protein expression of NLRP3 inflammasome-related factors to determine whether geniposide can improve myocardial injury by inhibiting the expression of NLRP3 and related proteins (Additional file 1: Fig.S1). Our data suggest that geniposide could decrease the levels of NLRP3, ASC, cleaved caspase-1, N-GSDMD, IL-1β, and IL-18 increased by MI/RI and H/R, both in vivo and in vitro. Additionally, we also found that an amount of NLRP3 was produced in the cytoplasm, and the production was apparently decreased after geniposide administration. Together, above data indicate that geniposide counteracts the MI/RI effect though reducing the activity of NLRP3 inflammasome and suppressing the activation of caspase-1 and IL-1β.

Importantly, activation of AMPK turns out to be a key mechanism to alleviate MI/RI [42, 43]. Previous research suggested that long-term inhibition of AMPK activity in transgenic mice model exerted enzyme activity deficiency, exacerbated cardiac dysfunction and cell apoptosis caused by MI/RI [44]. In the present study, geniposide simultaneously decreased the NLRP3 inflammasome and activated p- AMPK expression assisted geniposide’s ability to suppress inflammation and increased the viability of cardiomyocyte. After H/R procedure, cell membrane damage and LDH leakage increased, and NLRP3 inflammasome related factors were further increased when we suppressed the AMPK pathway by compound C, while AICAR reversed the damage caused by MI/RI or H/R. Previous studies also showed that AMPK signaling pathway and inflammasome play an important role in regulating systemic inflammatory reaction. Moreover, inhibiting AMPK activation induced by molecular inhibitors, such as compound C or sunitinib, and MCC950 inhibit NLRP3 inflammasome activity and mediate the opposite effect during MI/RI, which is consistent with our results [45–47]. While the direct interaction between AMPK and NLRP3 protein expression in MI/RI has not been reported yet. Our data indicated that AMPK as a key signaling pathway in the geniposide-mediated cardioprotective effect on the NLRP3 inflammasome during MI/RI. Furthermore, our research found geniposide protective effects of MI/RI were abolished by Compound C, which could inhibit functional of AMPKα. We also demonstrated our hypothesis that the suppression of NLRP3 was regulated by the activated AMPKα. The elevated level of NLRP3 was reversed by geniposide, while compound C attenuated the protective effect of geniposide on inflammation. In contrast, the combination of geniposide and AICAR further reduced the inflammatory level.

Previous reported that activated AMPK mediates energy metabolism and regulates mitochondrial homeostasis, which was closely related to NLRP3 activation [48]. Mitochondrial disorders are usually accompanied by activating and phosphorylating AMPK to avoid risk of cell bioenergy defects. Moreover, mitochondria play an important role in activating the generation of ROS in many pathological processes [49]. Activating AMPK is likely to be participated in cellular defense and resistance to oxidative stress initiated by mitochondrial ROS production [21, 50, 51]. Previous studies suggest that under various external stimulation (ischemia and hypoxia), the level of oxidative stress in cells increased sharply and upregulated the NLRP3 inflammasome [52, 53]. However, Trx is a major intracellular thiol reducing and reactive oxygen species (ROS) scavenging protein. In the present study, coimmunoprecipitation demonstrated that binding of TXNIP to Trx inhibits Trx activation and promotes oxidative stress. Under oxidative conditions, ROS accumulation facilitates TXNIP–Trx dissociation, thereby promoting the interaction between NLRP3 and TXNIP. NLRP3–TXNIP is required for inflammasome formation and activation. Therefore, TXNIP is postulated to be a key switch linking oxidative stress to inflammation. Moreover, TXNIP is a key protein in the ROS overproduction [54]. The translocation of TXNIP from the nucleus into the cytoplasm promotes binding to NLRP3 and mediates the activation of NLRP3 inflammasome or apoptosis signal-regulating kinase1 (ASK-1) which is related to cell apoptosis process [55].

Our DHE, SOD2 activity, and JC-1 results show that geniposide significantly inhibits the increase in ROS levels caused by MI/RI or H/R and effectively relieves oxidative stress. Moreover, geniposide reduces the level of TXNIP protein expression, but also weakens the interaction between TXNIP and NLRP3. Therefore, our study illustrates that the geniposide exerted cardioprotection and suppressed NLRP3 inflammasome might be heavily dependent on TXNIP, which is the functional interaction between AMPK and NLRP3, as it manifested a novel relationship between inflammation/ROS and energy homeostasis. Nevertheless, further studies are needed to investigate other mechanisms underlying the protective effect of geniposide in MI/RI. Here, geniposide played a significant myocardial protective effect in MI/RI model mice induced by coronary artery ligation, which can effectively combat MI/RI, while activating AMPK cell energy metabolism and suppressing the activation of NLRP3 inflammasome, one of its important mechanisms. This study provides experimental basis for the use of geniposide to prevent and treat cardiovascular diseases and proposes a new explanation of its mechanism of action.

## Conclusions

To our knowledge, this is the first study that provides strong evidence both in vivo and in vitro that therapeutic benefits of geniposide against MIRI largely depend on the activation of AMPK signaling and the inhibition of ROS/TXNIP/NLRP3 mediating inflammation and subsequent pyroptosis-related signaling pathways. These results not only elucidate the pathological mechanisms of MI/RI but also provide a pathway for future clinical drug inception and research.

## Supplementary Information

**Additional file1:** **Figure S1. **Specific number and proportion of mice that died/survived (included in the assay) after modeling between the different groups, as well as the chi-square test between the groups. 

## Data Availability

All datasets were used and/or analyzed during the current study are available from the corresponding author on reasonable request.

## Abbreviations

ACC: Acetyl-CoA carboxylase
AMPK: AMP-activated protein kinase
ASC: Apoptosis-associated speck-like protein containing a caspase-1 recruitment domain
CK: Creatine kinase
CK-MB: Creatine kinase-MB
Co-IP: Coimmunoprecipitation
DMEM: Dulbecco’s modified Eagle’s medium
FBS: Fetal bovine serum
FS: Fractional shortening
GEN: Geniposide
HE: Hematoxylin and eosin
IL: Interleukin
JC-1: 5,5′,6,6′-Tetrachloro-1,1′,3,3′-tetraethylbenzimidazolcarbocyanine iodide
LAD: Left anterior descending;
LDH: Lactic dehydrogenase
Lip2000: Lipofectamine^™^2000
LVEF: Left ventricular ejection fraction
LVFS: Left ventricular fractional shortening
LVEDd: Left ventricular end-diastole diameter
LVESd: Left ventricular end-systolic diameter
NLRP3: Nucleotide-binding domain, leucinerich-containing family, pyrin domain-containing-3
MI/RI: Myocardial ischemia reperfusion injury
N-GSDMD: The N terminal of gasdermin D
NRVMs: Neonatal rat ventricular myocytes
TTC: 2,3,5-Triphenyltetrazolium chloride
TXNIP: Thioredoxin-interacting protein
