# Supplementary material for: Geniposide suppresses NLRP3 inflammasome-mediated pyroptosis via the AMPK signaling pathway to mitigate myocardial ischemia/reperfusion injury
Source: Chin Med. 2022 Jun 17;17:73. doi: 10.1186/s13020-022-00616-5 (PMC9205109; doi:10.1186/s13020-022-00616-5)
Supplement: Supplementary file 1 — Additional file1: Figure S1. Specific number and proportion of mice that died/survived (included in the assay) after modeling between the different groups, as well as the chi-square test between the groups. [file 13020_2022_616_MOESM1_ESM.pdf]

A

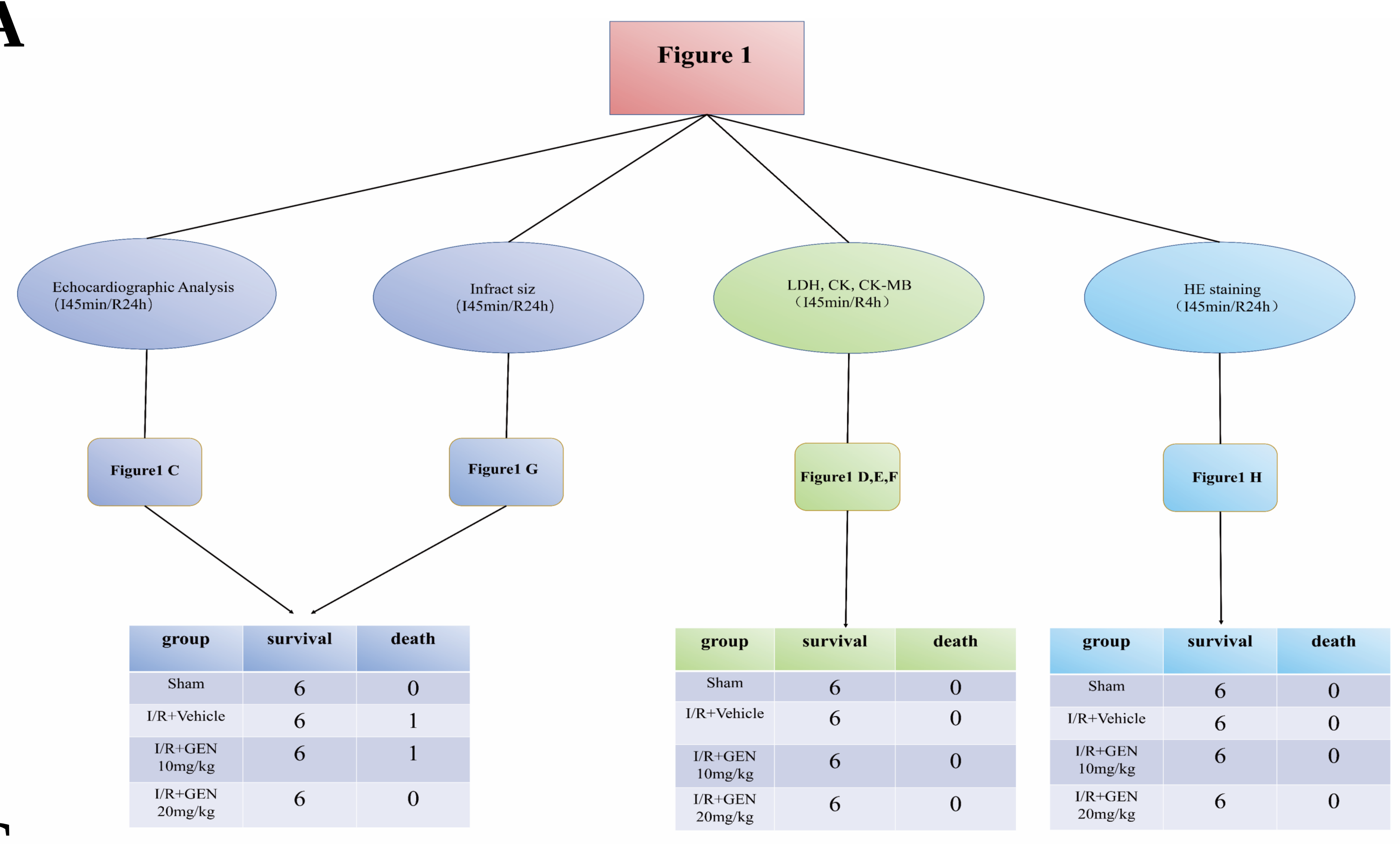

B

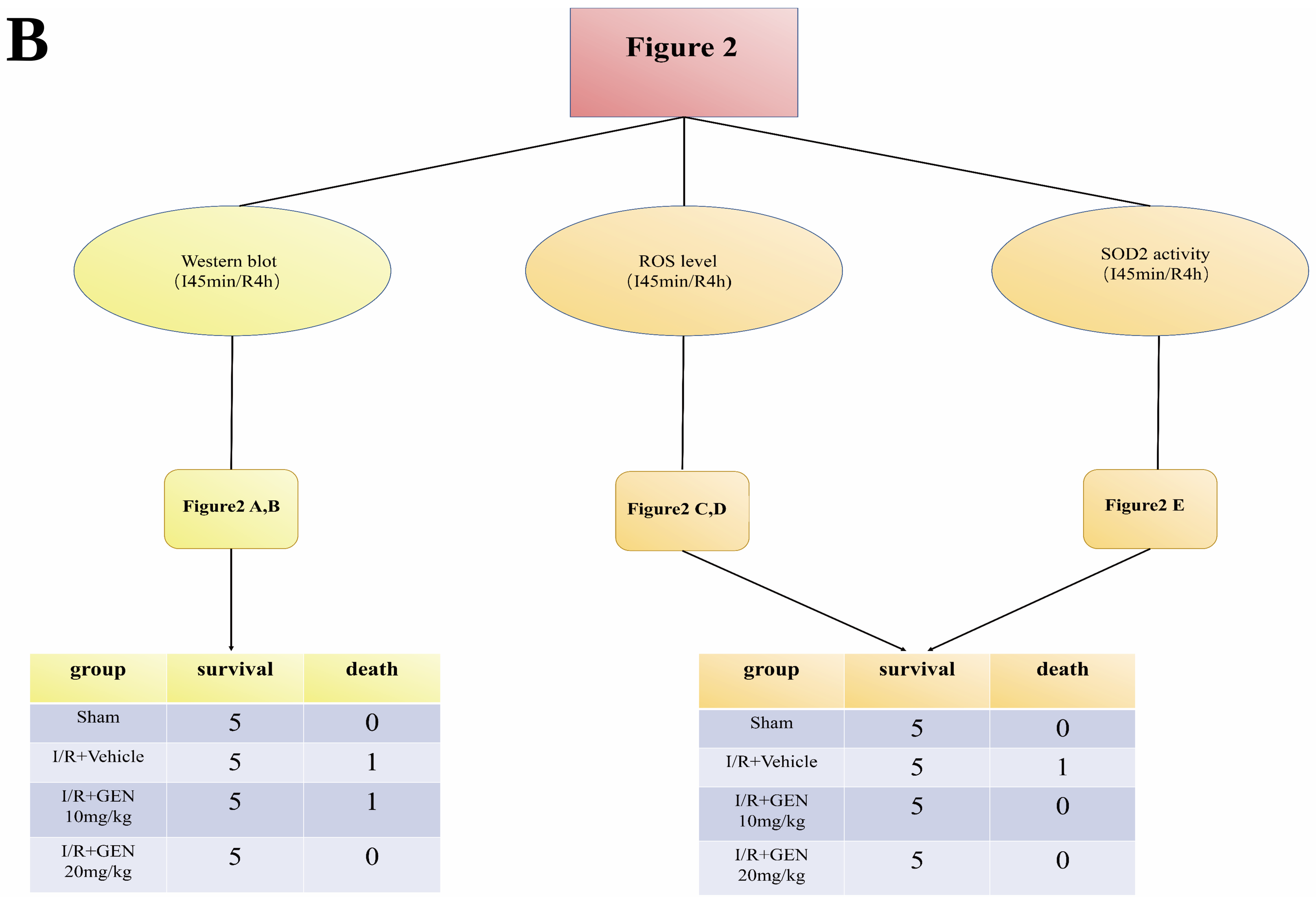

C

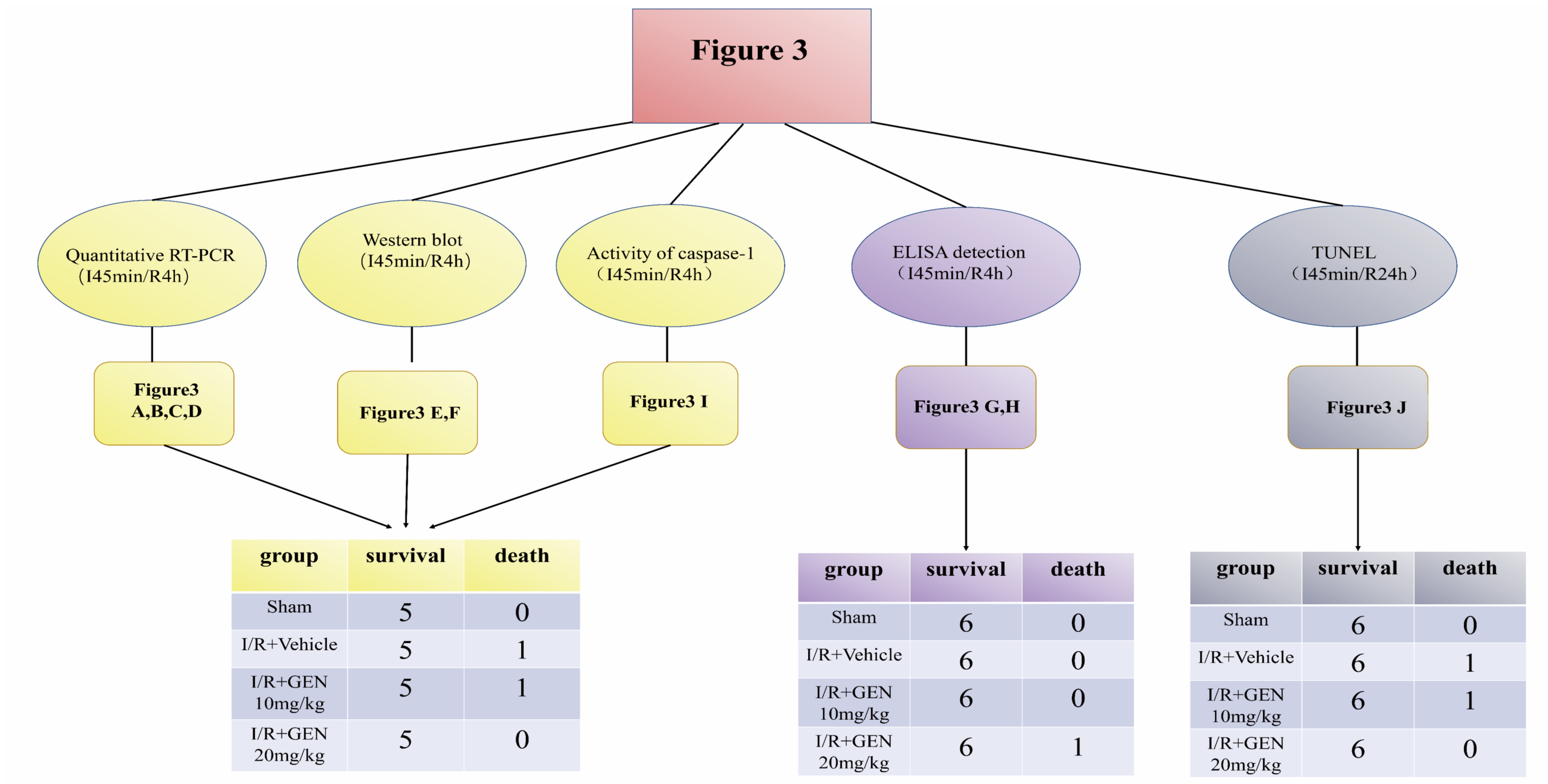

D

Group	Reperfusion for 4h	
	Survival	Death
Sham	22	0
I/R+Vehicle	22	2
I/R+GEN 10mg/kg	22	1
I/R+GEN 20mg/kg	22	1

Group	Reperfusion for 24h	
	Survival	Death
Sham	18	0
I/R+Vehicle	18	2
I/R+GEN 10mg/kg	18	2
I/R+GEN 20mg/kg	18	1

Group	Reperfusion(4h+24h)	
	Survival	Death
Sham	40	0
I/R+Vehicle	40	4
I/R+GEN 10mg/kg	40	3
I/R+GEN 20mg/kg	40	2

E

Items	Groups(n/%)				χ^2	*P*
	Sham	I/R+Vehicle	I/R+GEN 10mg/kg	I/R+GEN 10mg/kg		
**Reperfusion for 4h**					1.911	0.900[a]
Survival	22(100.0)	22(91.7)	22(95.7)	22(95.7)		
Death	0	2(8.3)	1(4.3)	1(4.3)		
**Reperfusion for 24h**					2.130	0.747[a]
Survival	18(100.0)	18(90.0)	18(90.0)	18(94.7)		
Death	0	2(10.0)	2(10.0)	1(5.3)		
**Reperfusion for (4+24h)**					3.913	0.292[a]
Survival	40(100.0)	40(90.9)	40(93.0)	40 (95.2)		
Death	0	4(9.1)	3(7.0)	2(4.8)		

Note: [a]: Based on Fisher exact test.

F

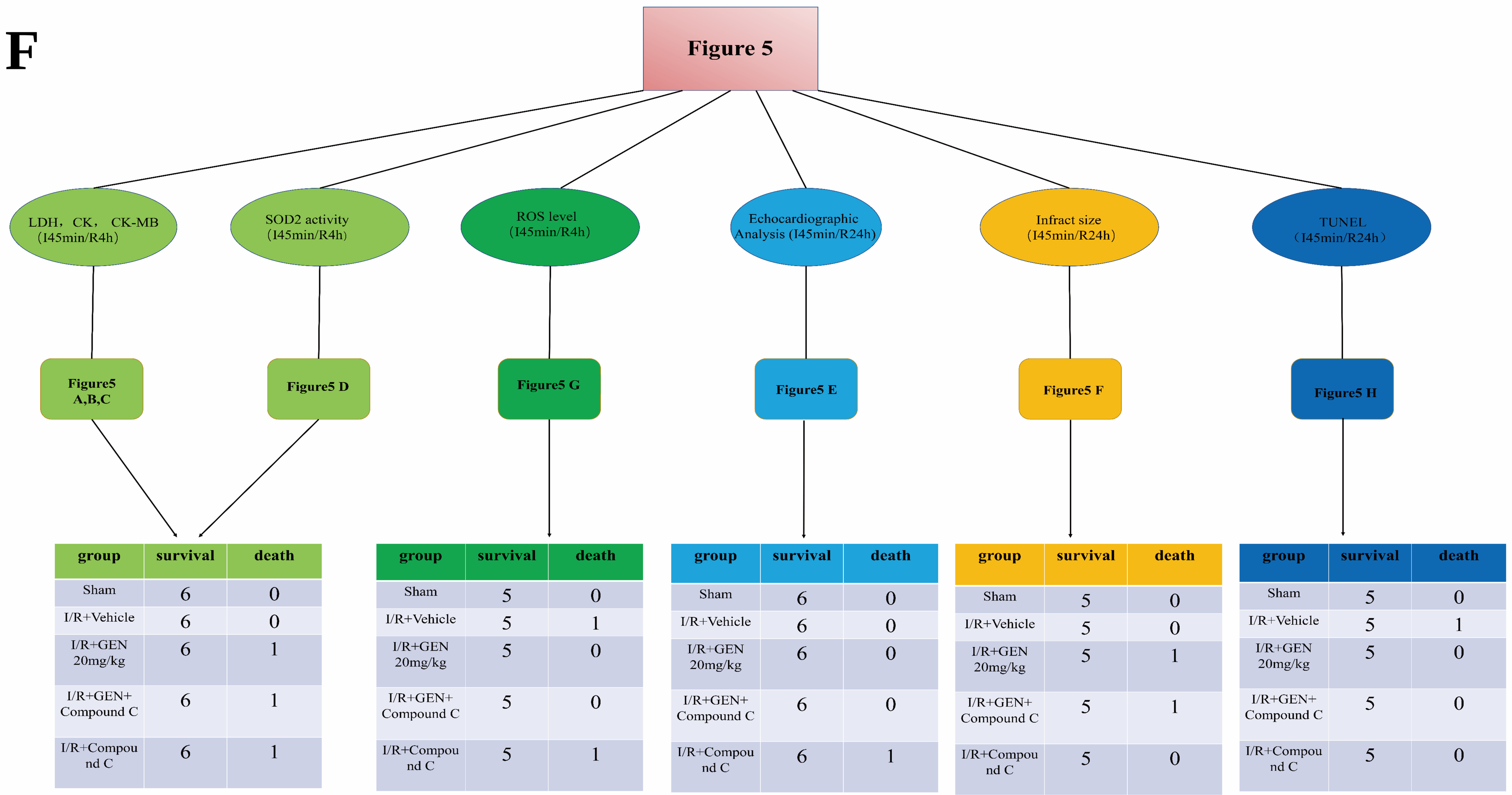

G

Group	Reperfusion for 4h	
	Survival	Death
Sham	11	0
I/R+Vehicle	11	1
I/R+GEN 20mg/kg	11	1
I/R+GEN+ Compound C	11	1
I/R+Compound C	11	2

Group	Reperfusion for 24h	
	Survival	Death
Sham	16	0
I/R+Vehicle	16	1
I/R+GEN 20mg/kg	16	1
I/R+GEN+ Compound C	16	1
I/R+Compound C	16	1

Group	Reperfusion(4h+24h)	
	Survival	Death
Sham	27	0
I/R+Vehicle	27	2
I/R+GEN 20mg/kg	27	2
I/R+GEN+ Compound C	27	2
I/R+Compound C	27	3

H

Items	Groups(n/%)					χ^2	*P*
	Sham	I/R+Vehicle	I/R+GEN 20mg/kg	I/R+GEN+ Compound C	I/R+ Compound C		
**Reperfusion for 4h**						2.017	0.955[a]
Survival	11(100.0)	11(91.7)	11(91.7)	11(91.7)	11(84.6)		
Death	0	1(8.3)	1(8.3)	1(8.3)	2(15.4)		
**Reperfusion for 24h**						1.627	1.000[a]
Survival	16(100.0)	16(94.1)	16(94.1)	16(94.1)	16(94.1)		
Death	0	1(5.9)	1(5.9)	1(5.9)	1(5.9)		
**Reperfusion for (4+24h)**						2.357	0.677[a]
Survival	27(100.0)	27(93.1)	27(93.1)	27(93.1)	27(93.1)		
Death	0	2(6.9)	2(6.9)	2(6.9)	2(6.9)		

Note: [a]: Based on Fisher exact test.

sFig.1 Specific number and proportion of mice that died/survived (included in the assay) after modeling between the different groups, as well as the chi-square test between the groups. A Total number of animals and death and survival included in Figure 1.**B** Total number of animals and death and survival included in Figure 2.**C** Total number of animals and death and survival included in Figure 3.**D** List the total number of animals and death and survival included in Figure 1-3. **E** Fisher exact test between the groups on the survival and death of animals at 4h and 24h after reperfusion, and the total survival and death of animals after reperfusion (4h+24h) respectively. **F** Total number of animals and death and survival included in Figure 5. **G** List the total number of animals included in Figure 5 and the number of dead and alive. **H** Fisher exact test between the groups on the survival and death of animals at 4h and 24h after reperfusion, and the total survival and death of animals after reperfusion (4h+24h) respectively.
